# A Trustworthy Key Generation Prototype Based on DDR3 PUF for Wireless Sensor Networks

**DOI:** 10.3390/s140711542

**Published:** 2014-06-30

**Authors:** Wenchao Liu, Zhenhua Zhang, Miaoxin Li, Zhenglin Liu

**Affiliations:** School of Optical and Electronic Information, Huazhong University of Science and Technology, No. 1037 Luoyu Road, Wuhan 430074, China; E-Mails: saintlawhd@gmail.com (W.L.); zzh373576922@gmail.com (Z.Z.); l.alveira@gmail.com (M.L.)

**Keywords:** WSN, PUF, DDR3, key generation, fuzzy extractor

## Abstract

Secret key leakage in wireless sensor networks (WSNs) is a high security risk especially when sensor nodes are deployed in hostile environment and physically accessible to attackers. With nowadays semi/fully-invasive attack techniques attackers can directly derive the cryptographic key from non-volatile memory (NVM) storage. Physically Unclonable Function (PUF) is a promising technology to resist node capture attacks, and it also provides a low cost and tamper-resistant key provisioning solution. In this paper, we designed a PUF based on double-data-rate SDRAM Type 3 (DDR3) memory by exploring its memory decay characteristics. We also described a prototype of 128-bit key generation based on DDR3 PUF with integrated fuzzy extractor. Due to the wide adoption of DDR3 memory in WSN, our proposed DDR3 PUF technology with high security levels and no required hardware changes is suitable for a wide range of WSN applications.

## 1. Introduction

WSNs are vulnerable to various attacks in hostile environments [1,2]. Within a typical node capture attack, an adversary can gain full control over the captured sensor nodes. Moreover, all contents of the non-volatile memory, including cryptographic secrets and other valuable data can be extracted through semi/fully-invasive physical attacks (e.g., micro-probing, reverse engineering *etc.* [3]). The cryptographic key leakage often leads to the failure of the whole security mechanism [4]. Therefore, a tamper-resistant and cost-efficient secure key provisioning scheme against node capture attack is very important to WSN.

Physically unclonable function (PUF) is a collection of functional constructions which meets a number of naturally identifiable characteristics such as randomness, uniqueness, physical unclonability, and possibly tamper-resistant [5]. The PUF presents a unique mapping between challenges (inputs) and responses (outputs) based on the device's hardware-intrinsic features. Those features are originated from the subtleties of the operating conditions as well as random variations that are imprinted into the device during the manufacturing process. Any physical attacks will destroy the inherent physical features with very high probability. Consequently, the response would also be destroyed.

There are several advantages over using PUF for secret key generation and storage. The PUF is uniquely linked to a given device such that one cannot reproduce it or manufacture a device with a precise key. In addition, the PUF avoids the need for storing secret keys in non-volatile memory as keys can be extracted from the device only when required. Moreover, PUF could be tamper-resistant, which is innate immunity to node capture attacks.

However, the PUF responses cannot be used directly as a cryptographic key. Due to the influence of environmental noises, the responses are likely to be slightly different for each evaluation for the same challenge. On the other hand, the PUF responses may not be uniformly distributed. To derive a unique and almost uniform key from PUF responses, a fuzzy extractor can be used [6].

### 1.1. Previous Work

Pappu *et al.* initially introduced the idea of PUF [7,8] based on the response (scattering) obtained when shining a laser on a bubble-filled transparent epoxy wafer. A number of experiments were performed to test the characteristics of the constructed PUF. The inter- and intra-distance measures were evaluated for the obtained Gabor hashes. This resulted in an average inter-distance *μ*_inter_ = 49.79% (σ_inter_ = 3.3%) and an average intra-distance *μ*_inter_ = 25.25% (σ_inter_ = 6.9%). The implement of optical PUF is rather laborious that a laser and a tedious mechanical positioning system are needed.

Gassend *et al.* proposed silicon PUF [9], which used manufacturing process variations in ICs with identical masks to uniquely characterize each IC. The statistical delay variations of transistors and wires in the IC were used to create a parameterized self-oscillating circuit and its frequency measurements are collected to characterize each IC. The integration of PUF into the underlying manufacturing variability of ICs makes it much more suitable for practical application.

Ring oscillator PUF, as introduced in [10], use a different approach toward measuring small random delay deviations caused by manufacturing variations. The output of a digital delay line is inverted and fed back to its input, creating an asynchronously oscillating loop. Due to random manufacturing variations in the circuit latency, the exact frequency will also be random and unique per device. They tested a ring oscillator PUF with division compensation on four FPGA devices obtaining *μ*_inter_ ≈ 10 × 10^−3^ and *μ*_inter_ ≈ 0.1 × 10^−3^ with measurements taken over a 25 °C temperature interval.

The flip-flop PUF was introduced by Maes *et al.* in [11]. The presented measurement results show that the entropy in the measured flip-flop power-up values is rather limited due to the presence of a strong bias, namely the strong preference of most flip-flops to power up with a zero value.

SRAM PUF was proposed in [12], originates from the mismatch of the symmetry of the memory cell, which is again caused by silicon manufacturing variability. The responses of SRAM PUF can be obtained from the power-up value of uninitialized SRAM cells. In [13], extensive experiments on SRAM PUFs were performed by collecting the power-up state of 8190 bytes of SRAM from different memory blocks on different FPGAs. The results show an average inter-chip Hamming distance of μ_inter_ = 49.97% between two different blocks and the average intra-chip Hamming distance of μ_intra_ = 3.57% within multiple measurements of a single block under typical environment and μ_intra_ < 12% for large temperature deviations.

The PUF properties for 28-nm process field-programmable gate arrays (FPGAs) are examined by Hori *et al.* in [14]. In their work, the performance of arbiter PUF and delay-based PUF are evaluated on Kintex-7 FPGAs on 10 SASEBO-GIII boards and Artix-7 on eight Nexys4 boards. A performances comparison with those on the 45-nmSpartan-6 and 65-nm Virtex-5 were analyzed. The results show that both APUFs and PL-PUFs on the 28-nm FPGAs demonstrated good properties that were no worse than those on 45- and 65-nm FPGAs. Therefore, the PUFs can be implemented using the state-of-the-art process technology and used for various security-sensitive applications.

Among all the proposed silicon PUF instantiations in literature, memory-based intrinsic PUF is the most promising solution for secure key provisioning scheme in WSNs. Memories are standard components in sensor nodes. Cryptographic keys can be generated without any change to the existing hardware architecture. DRAM PUF [15,16] utilizes the decay signature of the memory cells with one transistor/one capacitor structure. The manufacturing process introduces miniscule variations to the decay features of these cells. Keller [15] customized a DDR3 controller to disable the refresh of the DRAM cells for PUF usage. Since most sensor nodes are not reconfigurable, it is hard to reproduce the PUF response without the customized DDR3 controller. Rosenblatt [16] introduced a random intrinsic chip ID generation method using decay fails in 32 nm SOI embedded DRAM. The intrinsic feature was detected by adjusting the wordline low voltage of embedded DRAM. The adjustment is still hard to realize without changing the circuits of the sensor nodes. In general, existing DRAM PUF solutions are not applicable to resource constrained WSN without hardware modifications.

### 1.2. Our Contribution

To study the decay feature of DDR3, a proper test system has been developed and set up. The key generation skeleton based on DDR3 PUF is established, which is suit for WSN. The challenges are power-switch time as well as the address and offset of memory cells with logic 1 before power off while the responses are power-on value of those cells after specific decay interval. According the experimental result, an efficient prototype of 128-bit key generation based on DDR3 PUF in conjunction with fuzzy extractor is proposed. Some implementation specific details as well as the PUF key performance are discussed.

### 1.3. Paper Outline

The rest of this paper is organized as follows: Section 2 explores the decay feature of DDR3 and describes the key generation framework. The experimental setup and DDR3 decay characterization are presented in Section 3. The post-processing process for reliable key generation is discussed in Section 4. Section 5 concludes the paper.

## 2. DDR3 PUF Exploration

### 2.1. Decay Feature of DDR3

**Figure 1.**
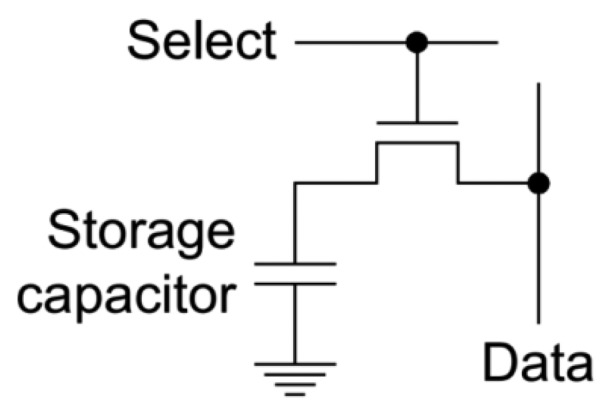
DRAM cells structure.

The foundation of DDR3 PUF lies in the underlying decay feature of DRAM cells. A DRAM cell consists of a capacitor connected by a pass transistor to the bit line as shown in Figure 1. The presence or absence of charge in the capacitor determines whether the cell contains a “1” or “0”. This charge would leaks off the capacitor due to the sub-threshold current of the cell transistor. The leaky nature varies among DRAM cells due to the miniscule variations introduced by manufacturing process. To prevent the charge leakage, a refresh operation is required which re-charges the capacitor to hold its value.

**Figure 2.**
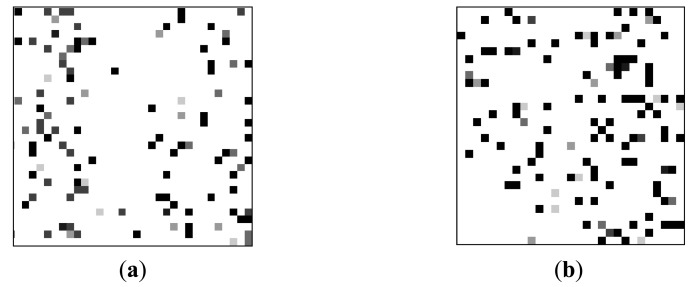
DRAM cells decay feature with power-switch interval time of 120 s: (**a**) DDR3 device 1; (**b**) DDR3 device 2.

To explore the decay feature, a proper test scheme has been developed to simulate typical WSN application scenarios in which the sensor nodes are often intermittently-powered. We write a “1” to every cell in a DRAM region, represented by the charge kept in the capacitors. Then, we power off the DRAM to terminate the refresh operation. After a pause, we cycle the DRAM power. Each cell is read and recorded as PASS if the charge has not leaked to logic “0” and FAIL if the charge has leaked to logic “0”. We repeat the above operations multiple times to minimize the influence of the environmental noises. To show decay feature of each DDR3 cells in an intuitional way, we transforms test data to a grayscale images illustrated in Figure 2.

As shown in Figure 2, the pixel position stands for the address of each DRAM cell. The decay feature is represented by the grayscale of the associated pixel which is equal to the total number of the failed test times divided by iteration times. The grayscale signifies the preference of decay feature in each cell. For example, a black pixel means the associated DRAM cell is always leaked to logic “0” during the interval time between power switching; while a white pixel means the leakage never happens during the interval. The decay feature between different DDR3 cells is different. This is hold for different DDR3 devices by comparing Figure 2a to Figure 2b. Thus the distribution of decay feature is unique.

The decay feature also varies with the interval time. If it is too short, almost no changes have occurred. If given enough time, the charges will vanish and no information can be extracted. Therefore, the optimal interval time should be chosen based on extensive profiling process. This innate decay feature enables DDR3 to be used as a PUF instantiation.

### 2.2. Key Generation Framework

The PUF responses cannot be used directly as a cryptographic key for two reasons. First, due to the influence of the existing environmental noises, the responses from the same DDR3 PUF could be slightly different each time. Second, the keys directly extracted from the DDR3 PUF responses are not uniformly distributed. Fuzzy extractor is used to as a necessary post-processing step of the PUF responses. The fuzzy extractor with an error correct code (ECC) module and a universal hash module work together in two phases: enrolment and key reconstruction. First, we introduce some terms [17] used in post-processing algorithm.

*Statistical distance*: Let *X* and *Y* be two random variables with range *U*. Then the *statistical distance* between *X* and *Y* is defined as:

(1) $$\Delta (X,Y)\equiv \frac{1}{2}\sum_{u\in U}\left|P\left[X=u\right]-P[Y=u]\right|$$

If two distributions have statistical distance of (at most) *ε* ≥ 0, they are *ε* – *close*:

(2) $$X\approx_{ɛ}Y\Leftrightarrow \Delta (X,Y)\leq ɛ$$

*Min-entropy*: Let *X be* a random variable. The Min-entropy of *X* is defined as:

(3) $$H_{\infty }(X)\equiv \underset{u\in U}{\min}\left\{-\log\left(P[X=u]\right)\right\}=-\log\left(\underset{u\in U}{\max P[X=u]}\right)$$

We say that *X* is a k-source if *H*_∞_ (*X*) ≥ *k*.

*Strong extractor*: Let the seed *U_d_* be uniformly distributed on {0,1}*^d^*. We say that a function Ext: {0,1}*^n^* × {0,1}*^d^* → {0,1}*^m^* is a (*k, ε*) strong random extractor if, for all random variables X in {0,1}*^n^* independent of *U_d_* with *H*_∞_(*X*) ≥ *k*:

(4) $$\left(\text{Ext}(X,U_{d}),U_{d}\right)\approx_{ɛ}(U_{m},U_{d})$$

where *U_m_* is uniformly distributed in {0,1}*^m^* independently of *X* and *U_d_*.

*Universal hash* [18]: Let *H* be a finite collection of hash functions from {0,1}*^n^* to {0,1}*^m^*, we say that *H* is universal hash if, for every *x,y* ∈ {0,1}*^n^* with *x* ≠ *y*:

(5) $$P_{h\in H}\left[h(x)=h(y)\right]\leq 2^{-m}$$

*Leftover hash lemma:* Let *X* be a random variable with universe *U* and *H*_∞_ (*X*) ≥ *k*. Fix *ε* >0. Let *H* be a universal hash family of size 2*^d^* with output length *m* = *k* − 2log(1/*ε*) + O(1) Define:

(6) Ext(x,h)=h(x)

Then Ext is a strong (*k, ε*/2) extractor with seed length *d* and output length *m*.

The Statistical distance between random variables *X* and a uniformly distributed *Y* reflects the randomness extent of *X*. The min-entropy provides a lower bound on the amount of randomness contained in the decay feature of the DDR3.The leftover hash lemma (LHL) shows how to construct extractors from universal hash families. Moreover, we can exactly calculate how many random bits can be derived from a response with *H*_∞_(*X*) ≥ *k* by fixing the statistical distance.

#### 2.2.1. Enrolment Phase

**Figure 3.**
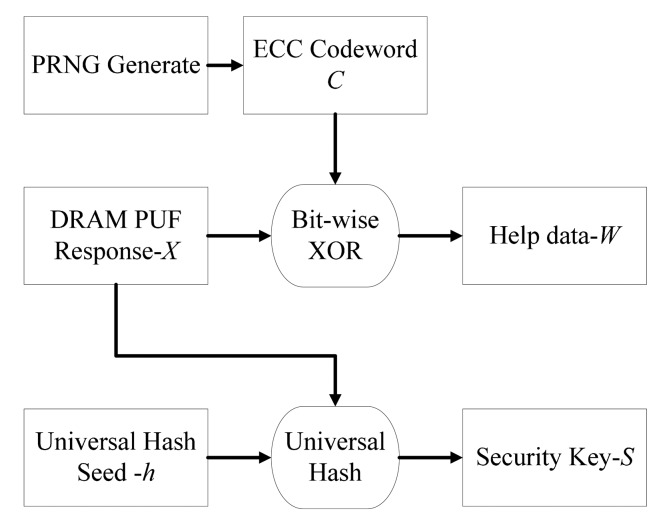
The flow chart of enrolment phrase.

The flow chart of enrolment phase is shown in Figure 3. Responses *X* measured from DDR3 decay as well as a seed *h* are sent to the universal hash module to get an almost uniformly distributed security key *S*. At the same time, a help data *W* is derived from bitwise XOR operation between the response *X* and ECC codeword *C*. The output of a PRNG Generate with uniform distribution is encoded to the ECC codeword *C*. The help data *W* and seed *h* will be saved and used in the key reconstruction process later.

The mathematical expression of enrolment phase is shown in Equation (7):

(7) $$\{\begin{array}{c} X⊕C=W\\ \{X,h\}\overset{\text{Universal Hash}}{\to }S \end{array}$$

Our goal is to extract almost uniformly distributed key from a response. The response *X* can be considered as a random variable due to the unpredictability. Our assumption is that we do not know the exact distribution but we know it comes from some class *D* of distributions. We would like *D* to be as general as possible, while still being able to extract good random bits.

The storage of the universal hash seed *h* is safe as it is independent to security key *S*. There is certain risk to store the helper data *W*.When the attacker get the *W*, the response *X* might be extracted by performing bitwise XOR operation between *W* and all possible ECC codeword. However, if the min-entropy of random ECC codeword is much larger the response, the leakage is safe. Thus those data can be stored in non-volatile memory without being encrypted. A more detailed procedure will be discussed in Section 4.

#### 2.2.2. Key Reconstruction Phase

**Figure 4.**
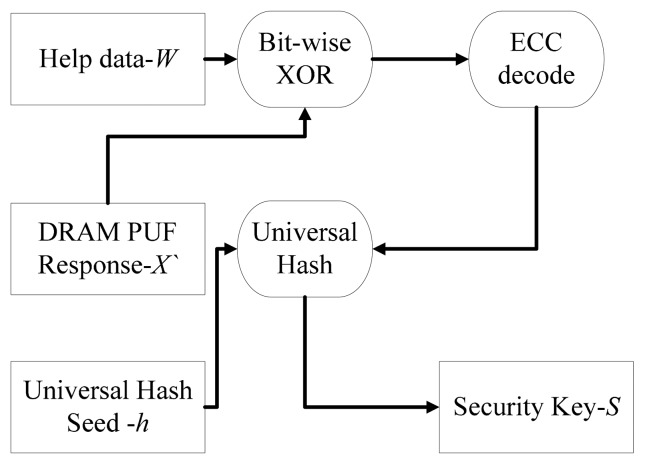
The flow chart of key reconstruction phrase.

The flow chart of key reconstruction phase is shown in Figure 4. A new response *X′* which might be slightly different from the response in enrolment phase is measured. Then, the bitwise XOR operation is performed between *X′* and the helper data *W*. An ECC decode module is applied to the result. The ECC output as well as the seed *h* is input to universal hash module. Finally, the security key *S* is reproduced.

The mathematical expression of the key reproduce phase is described in Equations (8):

(8) $$\{\begin{array}{c} X\prime ⊕W=C\prime \\ C\prime \overset{\text{ECC decoed}}{\to }C\\ e=C\prime ⊕C\\ X\prime ⊕e=X\\ \{X,h\}\overset{\text{Universal Hasha}}{\to }S \end{array}$$

An error version ECC codeword *C′* is calculated by bitwise XOR operation between *X′* helper data *W*. Then an ECC decode procedure is performed to correct errors in *C′*. If the error correct ability of ECC is stronger compared with the difference between *X′* and *X*, the ECC codeword *C* used in enrolment phase can be decoded. An error vector *e* is equal to *C* bitwise XOR with *C′*. *X* can be retrieved by bitwise XOR between *e* and *X′*. The security key *S* can be reproduced by input the retrieved *X* as well as the seed *h* into universal hash module.

## 3. Experimental Setup and Results

### 3.1. Metrics

To quantify the experimental results, several metrics are defined. The DDR3 PUF response is described in the form of binary string (0,1)*^n^*. Hamming distance is equal to the number of positions at which the two responses are different in terms of binary string. Normalized Hamming distance is equal to Hamming distance divided by the length of the response binary string times 100%.

Normalized Inter-Hamming distance is equal to normalized Hamming distance between two responses obtained from different DDR3 PUF. This measure expresses how easy it is to distinguish two DDR3 based on their decay feature. In general, it measures the uniqueness of the PUF responses with respect to the entire device population. Its value should be as close to 50% as possible.

Normalized intra-Hamming distance is equal to normalized Hamming distance between two responses obtained from the same DDR3 PUF. This measure reflects the degree of similarity between the two responses. Its value should be much small than normalized Inter-Hamming distance.

Decay rate is the number of “0” in a response binary string divided by the length of the response binary string times 100%.

Normalized min-entropy is equal to the min-entropy obtained from responses of the same DDR3 PUF divided by the length of the response binary string. The min-entropy is calculated by the method in [12]. Normalized min-entropy is a measure of the randomness in the responses.

### 3.2. Experimental Platform and Setup

**Figure 5.**
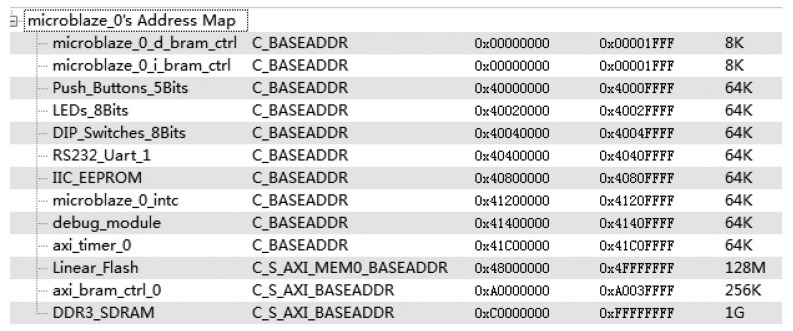
System memory map of BIST project.

The feasibility of DDR3 PUF is validated through experiments on a Xilinx VC709 evaluation board. The decay feature of two Micron DDR3 SODIMM and two Kingston DDR3 SODIMM is tested. The DDR3 is labeled as M1, M2, K1 and K2 respectively. The Built-In Self-Test (BIST) [19] example project in Xilinx EDK with DDR3 IP core can be used to access the DDR3 devices. The system memory map of BIST project is shown in Figure 5.

We first write a 1 to every cell from 0xD000 0000 to 0xD002 0000 of the DDR3. Then, we power off the evaluation board to terminate the memory refresh. After a pause, we power on the board. Each cell from 0xD000 0000 to 0xD002 0000 is read out. The experiments are repeated under 8 different intervals between power-switches. The specific interval time and experiment repeat times are list in Table 1.

The mean values of decay rate, normalized inter- and intra-Hamming distance and normalized min-entropy over 50 times experimental results at each intervals are analyzed.

### 3.3. Decay Rate

**Figure 6.**
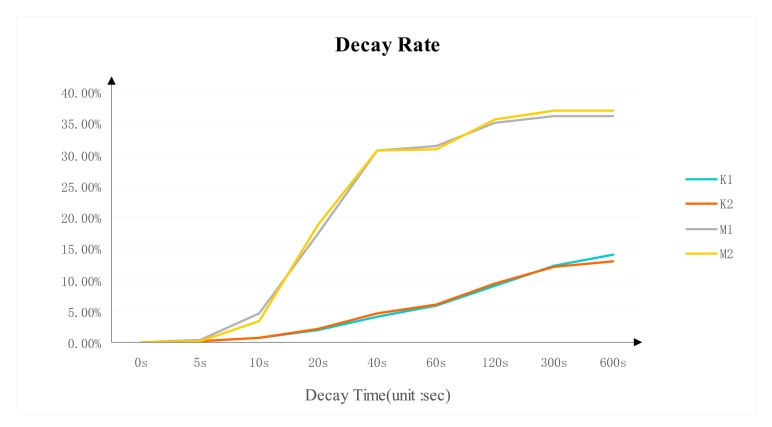
Decay rate for different DDR3 SODIMM.

The decay rate of DDR3 is shown in Figure 6. It displays significant differences between the two brands in decay trend and decay speed. The decay rate from the same DDR3 brand and model is almost identical. The Micron DDR3 decayed quickly after 10 s and the decay rate becomes stable around 35% after 120 s. The decay trend of Kingston DDR3 is relatively more stable than the Micron's. The decay rate is around 12% after 300 s.

### 3.4. Normalized Inter- and Intra-Hamming Distance

**Figure 7.**
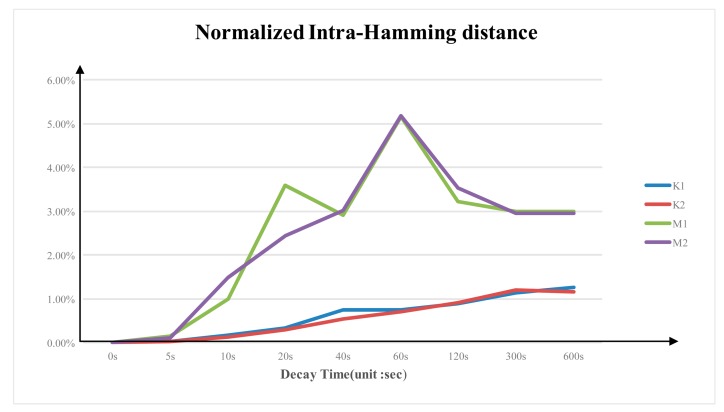
Normalized intra-Hamming distance for 4 DDR3 devices.

Figure 7 shows the average normalized intra-Hamming distance of the same DDR3 from multiple experiments at different power-switch time. Similar to the decay rate, the intra-Hamming distance rate varies between different brands. The average normalized intra-Hamming distance of Micron DDR3 is larger than that of the Kingston DDR3. Micron DDR3's average normalized intra-Hamming distance is around 3.6%; while Kingston DDR3's intra-Hamming distance rate is around 1.3%.

**Figure 8.**
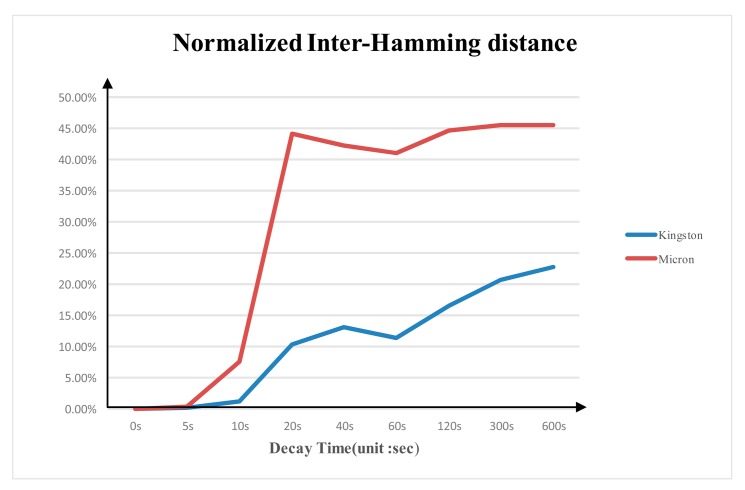
Normalized inter-Hamming distance between DDR3 devices with the same brand.

Figure 8 shows the average normalized inter-Hamming distance between different DDR3 with the same brand from multiple experiments at different power-switch time. The average normalized inter-Hamming distance of Micron DDR3 is larger than that of the Kingston DDR3. The average normalized inter-Hamming distance between two Micron DDR3s is around 45% and around 23% for the Kingston.

According to the test results, we validate the unique of DDR3 PUF with the fact that the average normalized intra-Hamming distance is much smaller than the inter-Hamming distance rate. By choosing the proper ECC code with error correction ability between the intra-Hamming distance rate and the inter-Hamming distance rate the DDR3 PUF response used in enrolment phase can be retrieved from the measured response.

### 3.5. Normalized Min-Entropy

**Figure 9.**
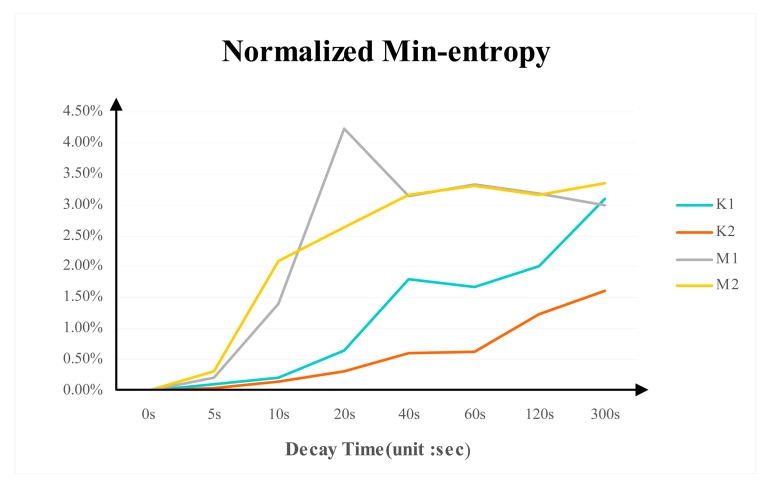
The normalized min-entropy for the tested DDR3 memories at different power-switch time.

Figure 9 shows the normalized min-entropy for the tested DDR3 memories at different power-switch time. The trend of normalized min-entropy is stable when the decay trend is stable. Micron DDR3's normalized min-entropy is around 3.3% and K1 normalized min-entropy is about 3% while the value for K2 is around 1.5%.

Normalized min-entropy quantifies the randomness of the PUF responses. The result shows that the DDR3 PUF is not uniformly distributed. In order to generate a 128-bit uniform key, the Min-entropy of DDR3 PUF should be at least 128. This means the number of cells used in DDR3 PUF is equal to 128 divided by normalized min-entropy.

## 4. Key Generation Prototype

The key generation prototype mainly focuses on determining the proper challenges of the PUF as well as the parameters of fuzzy extractor.

### 4.1. Challenges

According the experimental results, the decay rate of Micron 1 DDR3 will be stable after 120 s. Thus the power-switch time is equal to 120 s. The offset of memory bits is equal to the target key length divided by the min-entropy per bit that is about 3878. We set the offset to 4080 with a margin. By using this challenge, a response in the form of a 4080-bit binary string will be obtained in each measurement. The min-entropy of the 4080-bit response is about 134.64.

### 4.2. ECC Parameters

The length of ECC codeword is the same as the response length. The BCH forms a large class of powerful random error-correcting cyclic codes [20]. In order to simplify the process in encode and decode the 4080-bit response is divided into 16 groups with 255-bit per group. Some BCH code with block length 255 are listed in Table 2.

We randomly choose 16 BCH (255, 37, 45) codewords. The error correct ability of each codeword is 17.64% which is between the normalized intra-Hamming distance and normalized inter-Hamming distance of Micron 1 DDR3. Moreover, the codewords can be deemed as a random variable with 14.5% normalized min-entropy which is larger than the value of response. This implies the bitwise XOR operation between the response and an ECC code will not leak the entropy of the response.

### 4.3. Universal Hash Parameters

**Figure 10.**
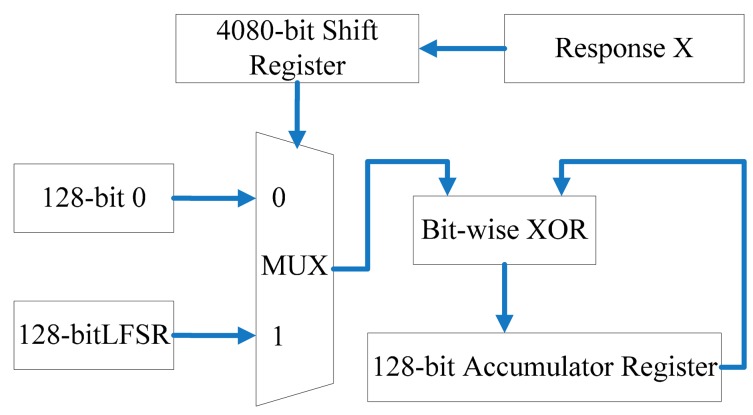
The structure of universal hash.

The Toeplitz matrix is a universal hash instantiation [21]. It can be represented by a linear-feedback shift register (LFSR). The Toeplitz matrix transforms the 4080-bit response to a 128-bit almost uniform key as shown in Figure 10.

The primitive polynomial for the 128-bit LFSR is:

(9) $$f(x)=1+x^{32}+x^{47}+x^{58}+x^{90}+x^{121}+x^{128}$$

The key can be calculated as:

(10) key=m(1)⊕m(2)⊕⋯⊕m(i)⊕m(4080)

where *m*(i) switches between a 128-bit 0 or the content of the 128-bit LFSR depending on the value at position i of the response binary string. The *m*(1) is the seed of the LFSR.

According to [6] the output key length, the min-entropy of input response and the statistical distance between the key and universal distribution on {0,1}^128^ should satisfy the equation below:

(11) m=k−2log(1/ɛ)+2

The statistical distance of 128-bit key between uniformly distributed is about 0.05 with *m* = 128 and *k* = 134.64.

Several keys have been generated from the other responses of the Micron 1 DDR3. After the universal hash process the normalized min-entropy in the key is about 70.7%, which is much bigger than its value of the responses before process.

## 5. Conclusions

In this paper we use the decay feature of DDR3 to build a PUF optimized for WSN key generation. Several evaluation metrics such as decay rate, normalized inter- and intra-Hamming distance and Min-entropy per bit are used to evaluate DDR3 PUF. The decay rate will be stable after some power-switch time. The unique decay feature is demonstrated with the fact that the intra-Hamming distance rate is much smaller than the inter-Hamming distance rate. The Min-entropy per bit of decay feature is acceptable for PUF usage The challenges are power-switch time as well as the address and offset of memory bits with logic 1 before power off while the responses are power-on value of those bits after specific decay interval. We construct a fuzzy extractor to eliminate the influence of the existing environmental noises and make key almost uniformly distributed. The parameters of fuzzy extractor are calculated according to the experimental result. A prototype of 128-bit key generation based on DDR3 PUF with integrated fuzzy extractor is presented. A 128-bit key is extracted from 4048-bit DRAM cells. Future works may involve environmental testing and large scale evaluation with more sample chips. With tamper-resistance at zero silicon cost and wide adoption in sensor nodes, DDR3 PUF is a promising key provisioning solution for various WSN applications.

## Figures and Tables

**Table 1.** Experimental Setup.

**Group ID**	**Interval Time (unit: s)**	**Repeat Times (unit: repeat times per each DDR3 device)**
1	5	50
2	10	50
3	20	50
4	40	50
5	60	50
6	120	50
7	300	50
8	600	50

**Table 2.** Some BCH code with block length 255.

**n**	**k**	**t**	**Error Correct Ability**	**Normalized Min-Entropy**
255	63	30	11.76%	24.71%
255	55	31	12.16%	21.57%
255	47	42	16.47%	18.43%
255	45	43	16.86%	17.65%
255	37	45	17.65%	14.51%
255	29	47	18.43%	11.37%
255	27	55	21.57%	10.59%
255	13	59	23.14%	5.10%
255	9	63	24.71%	3.53%
